# Utilization of mobile health applications and determinant factors among health science students at Debre Markos University, northwest Ethiopia in 2022

**DOI:** 10.1371/journal.pone.0275689

**Published:** 2023-07-13

**Authors:** Gizaw Hailiye Teferi, Maru Meseret Tadele, Getaye Tizazu, Zegeye Regasa Hordofa, Aynadis Worku Shimie, Bayou Tilahun Assaye, Andualem Fentahun Senishaw, Sefefe Birhanu Tizie

**Affiliations:** Health Informatics Department, College of Health Science, Debre Markos University, Debre Markos, Ethiopia; University of Science and Technology of Fujairah, YEMEN

## Abstract

**Background:**

High penetration of smartphones and ownership among the young generation increased the utilization of mobile health applications for health management among university students more than ever. Compared to other health service provision mechanisms mobile health service has higher advantages in promoting a healthy lifestyle since it is not limited to time and space. Even though there are numerous studies conducted in the area of mobile health utilization, this phenomenon is not well studied among university students in Ethiopia, Hence this study aims to determine the level of mobile health applications utilization among health science students.

**Method:**

A cross-sectional study was carried out at Debre Markos University from April 05 to June 25, 2022 among health science students. For this study, 423 students were recruited from health Science College using a systematic sampling method. The data was gathered using a self-administered questionnaire. Using SPSS version 26 software descriptive analysis, bivariate and multivariable logistic regressions were performed. A P-value of .05 at a 95% confidence interval was considered statistically significant. The questionnaire’s validity was determined by expert opinion, and by calculating its reliability using cronbach alpha (α = .78).

**Result:**

This study revealed that more than half 59% (144/244) of the study participants have installed and utilized 1–3 health-related apps. Among the students who have mobile health applications 38.6% utilize mobile health application several times a day while only 2.1% utilized rarely. Usage history shows that 40% of the study participants utilized the applications for a year, while 15.4% of the students utilized them for more than 2 years. Easiness of applications (AOR = 4.8, 95%CI: [2.1–11.2]), skill AOR = 4.2, 95%CI: [3.8–12]) and trust (AOR = 2.8, 95%CI: [1.6–4.8]) were among the factors that were associated with mobile health applications utilization. Students were asked about the barriers that limit the use mobile health applications and self-reported barriers includes Lack of knowledge and awareness of application benefits, and Lack of adequate time to use applications.

**Conclusion:**

The study shows that mobile health applications utilization is moderate relative to previous studies. Mobile health application developers for the young generation should consider healthy lifestyle related applications like fitness and weight loss apps.

## Background

In recent years, ownership of smartphones is drastically increasing in Ethiopia. This in turn facilitated the use of mobile phones to access health related information in addition to formal health care service [1].

Trends show that considerable healthcare services have been fully shifted to smartphones. Since the concept of mobile health (mHealth) was first coined in 2005 [2].

Compared to other health service provision mechanisms like web-based health services delivered from desktops and laptops, m-Health services have the advantage of interacting with individuals with greater frequency and flexibility, without being limited by time and place [3, 4].

Mobile health applications have revolutionized the way health science students’ access and learn about health-related information [5]. These apps provide easy access to study materials, textbooks, educational videos, and podcasts, making learning more accessible than ever before. Additionally, mobile health applications are regularly updated with new information, allowing students to stay informed about the latest developments in medical research and news in real-time [6, 7].

Furthermore, some mobile health applications offer interactive case study discussions and simulate medical scenarios, enhancing clinical knowledge and experience. This feature is particularly useful for students who want to gain practical experience before entering the workforce [8].

Statistics shows that there were about 53 thousand health apps on Google play at the last quarter of 2021 [9]. Among mobile health applications available on the market, the vast majority is designed for consumers [10]. Mobile phone based health apps developed for various aspects of healthcare, such as promoting healthy lifestyles, to provide assistance with diagnoses at the point of care, and improving patient care following treatment [11, 12]. By improving patient provider communication, health condition monitoring health information access, and enabling consumer’s self-care, mobile health applications could reduce rising healthcare costs [13, 14].

The high penetration of smartphones, interest and capability of customer in involvement of personal health has increased the utilization of mobile based health apps, this in turn increased the impact m-health on patients [15].

Understanding of which types of mobile health applications consumers are ready to use, as well as what barriers exist for the specific technologies are required to design and develop health related technologies [16].

Different studies conducted in field of mobile health apps utilization among University students reported inconsistent result. Recent study conducted among health science students in Saudi Arabia reported that 56% of the study population own smartphone health apps and about 28% the participants used the application for six months, 18% used the application for 7 to 12 months, 20.6% used the application for one to two years, and 17% utilized the application for more than two years [17]. The other study conducted in United Kingdom shows that, 79.8% of students had a health-related application and the most prominent users are who own iphone operating system(IOS) than other operating systems [18]. A cross-sectional study from United States of America revealed that 58% of the mobile phone owners had health related applications highlighting fitness and caloric intake applications were the most commonly downloaded and utilized [19]. A study conducted in Greece found that 57.7% of medical students had one to five mobile health applications on their mobile devices [20].

A study from Saudi Arabia, reported inconsistent use of health-related mobile applications. From the study participants, 89.1% of users reported they had a health-related application. Approximately 73% were occasional users of health application and only 27% reported using health applications application at least once a day [21].

The utilization of health-related apps is influenced by various factors, and one of the primary determinants that shape user behavior towards mobile health application usage is the perceived usefulness of the app [22, 23]. Recent studies revealed that user friendly interface and easiness of the apps as determinant factor for utilization of the apps among university students [24, 25]. The most significant obstacles for the adoption of health apps were the security concerns regarding personal health data, the intricacy of the apps, and the absence of recommendations from instructors [17, 26, 27]. Previous study reported that cost of the health application was one of the barriers that limit the utilization of mobile health application among college students [17].

Despite the widespread availability and recognition of mobile health applications (MHA) as a useful tool for healthcare management, there is limited knowledge about the level of adoption of MHA among health science students. This lack of information hinders the potential benefits of using technological innovations in managing health conditions and improving healthcare delivery. Therefore, understanding the factors that contribute to or hinder the adoption of MHA among health science students is crucial to promote and enhance their utilization of these apps. Even though a number of studies were conducted in the area of mobile health applications utilization among health care professionals in Ethiopia [28–31], limited studies were done among health and medical students, Therefore this study is conducted to determine the of level health related mobile applications utilization, to identify associated factors and The barriers that could hinder mobile health application utilization.

### Objective

The primary objective of this research was to evaluate the extent of mobile health application usage, identify factors that influence its utilization, and determine potential obstacles that may impede its adoption.

## Method

### Study area and design

A cross-sectional study was conducted among Debre Markos University health science college students from April 05 to June 25. Debre Markos University is one of the well-known Universities in Ethiopia located in northwest Ethiopia 300km from the capital city. The University has three campuses and 29,619 students and 1158 active academic staff. Since the sample size depends on the study type and the nature of the population under investigation we used a single population proportion formula with finite population correction [32], a 95% confidence level, since there was no previous study conducted a proportion of mobile health apps utilization 50% was taken [33], relative precision of 5%, and 10% non-response rate.

Samplesizen=(Zα2)2xp1−Pd2,(n)=(1.96)2x0.51−0.50.052=384.2+384.2*10%=422.616

Where;

- n = estimated sample size
- p = single population proportion (50%).
- Z_α/2_ = is value of standard normal distribution (Z-statistic) at the 95% confidence level (α = 0.05) which is 1.96,
- d = is the margin of error 5% (0.05)

Hence the total sample size was computed to be 423. A stratified sampling design with the proportional allocation technique was performed for each department. To select study participants from each department, systematic sampling technique was used.

### Inclusion criteria

Health science students who are enrolled at Debre Markos University in the academic year 2022 and are in their second year or above, and who own and use mobile devices such as smartphones and tablets, were included in this study.

### Study variables and operational definitions

#### Outcome variable

The dependent variable is utilization of mobile health application among health science students.

#### Independent variables

The independent variables were socio-demographic factors (age, sex, department, year of study, smartphone type, and health app availability), system-related factors (perceived ease of use), organizational factors (infrastructure), and behavioral factors (technical skill, trust).

#### Operational definition

Utilization of mobile health application refers to the frequency and extent to which an individual uses a software application installed on their mobile device to access health-related information, track their health status, monitor their fitness level, receive reminders for medication or appointments, communicate with healthcare providers, and engage in other health-related activities [34].

Perceived ease of use: The level of difficulty or simplicity in using a mobile health application or technology for health-related purposes. It is a measure of how easy it is for an individual to navigate and interact with the mobile health technology, including tasks such as accessing information, inputting data, and receiving feedback [35].

Technical skill: The ability to effectively use and navigate mobile health technologies, such as mobile applications, wearable devices, and telemedicine platforms, for the purpose of collecting and analyzing health-related data [36].

Trust: is a degree to which individuals believe that the mobile health technology they are using is reliable, secure, and effective in providing accurate health information and facilitating communication with healthcare providers [37].

#### Data collection and quality control

A structured, self-administered questionnaire was used to collect the data. The tool was taken from a previous study and adapted to our context [17]. Before the main data collection pilot study was done among 21 students at Technology College in Debre Markos University and a reliability test was done using Cronbach’s alpha to test the validity and internal consistency of the tool. Based on the pretest test modification was made to the questionnaire. The questionnaire consists of 21 items encompassing application use, the purpose of applications used, the type of applications used, barriers that hinder apps usage and factors. In addition to this, there was an open-ended question that request to indicate recommended health applications for others to use. Students from the Public Health, Nursing, Medicine, Midwifery, Pharmacy, Health informatics, Radiology, and medical laboratory participated in the study. Students were invited to participate during their first class in the morning for 45minutes. Training was provided for the data collectors for two days about the objective of the study, study participant’s rights and data collection procedures. Supervisors made close supervision on participant’s right and data quality.

#### Data analysis

The data entered using Ep-info version 13 software. Data analyzed with SPSS Version 26. To represent socio-demographic characteristics of the study participants descriptive analyses were performed and to determine the level of mobile health utilization. Bivariate logistic regression was conducted to identify the effect of each independent variable on the outcome variable at 95% confidence level, variables with p-value of .02 were considered for multivariable logistic regression. Multivariable analysis was conducted to identify notable variables associated with mobile health use and utilization. Adjusted odds ratios were used to measure the association of dependent and independent variables, 95% confidence intervals, and P-value was calculated to evaluate statistical significance.

#### Ethical considerations

This study followed all ethical standards for carrying out a research. Ethical approval was received from Debre Markos University ethical review Board. Permission to access participants was obtained from each department head. In addition, all participants signed an informed consent form. Participation in the study was voluntary, and no incentive was given.

## Result

### Socio demographic characteristics of the study participants

Of 423 distributed questionnaires, 405 valid responses were received with a response rate of 95%. Most of the study participants were within the age group of 20–25 years. Most of the study participants were male, 219(54%). Regarding specialization, about 15.5% of the study participants were from the public health stream, whereas 13.1% were from the midwifery stream. Most of the study participants have an android operating system smartphone, 73.6%. The overall percentage of students who currently own a health-related application was 60.2% Table 1.

**Table 1:** Socio-demographic characteristics of the study participants (N = 405).

Variable	Category	Frequency	Percentage
**Gender**	Male	219	54
	Female	186	46
			
**Age**	Under 20 years	61	15
	20–25 years	302	74.6
	Over 25 years	42	10.4
			
**Stream**	Public Health	63	15.5
	Pharmacy	27	6.7
	Nursing	50	12.3
	Health Informatics	33	8.1
	Medicine	52	12.8
	Medical Laboratory	34	8.4
	Midwifery	53	13.1
	Radiology	16	3.9
	Environmental Health	45	11.1
	Nutrition Science	32	7.9
**Smartphone type**	Android	296	73.6
	IOS	71	17.5
	Others	36	8.9
**Health related app ownership**	Yes	244	60.2
	No	161	39.8

Others: include operating systems like Windows mobile, Blackberry, and Ubuntu

### Utilization of mobile health applications among health science students (N = 244)

**Fig 1:**
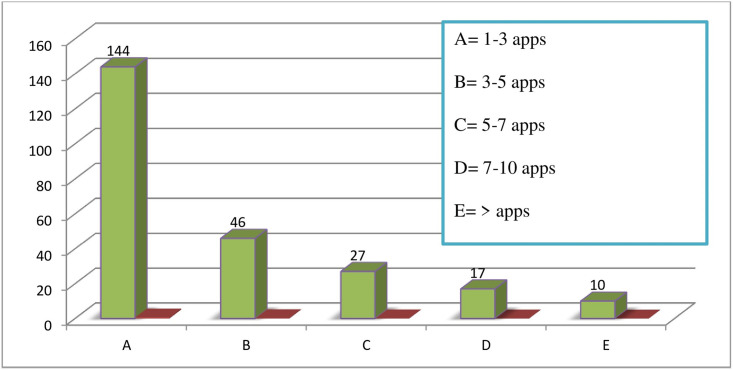
Number of health related applications owned by students (N = 244).

The utilization level of mobile health apps among health science students was found to be 60.5% (95% CI: [56.6–65.5]). The study showed that a majority of the participants, 59% (144), had installed and used 1–3 health-related apps on their smartphones for various health-related reasons. However, only 4.3% of the students had more than 10 health applications installed on their devices Fig 1.

**Fig 2:**
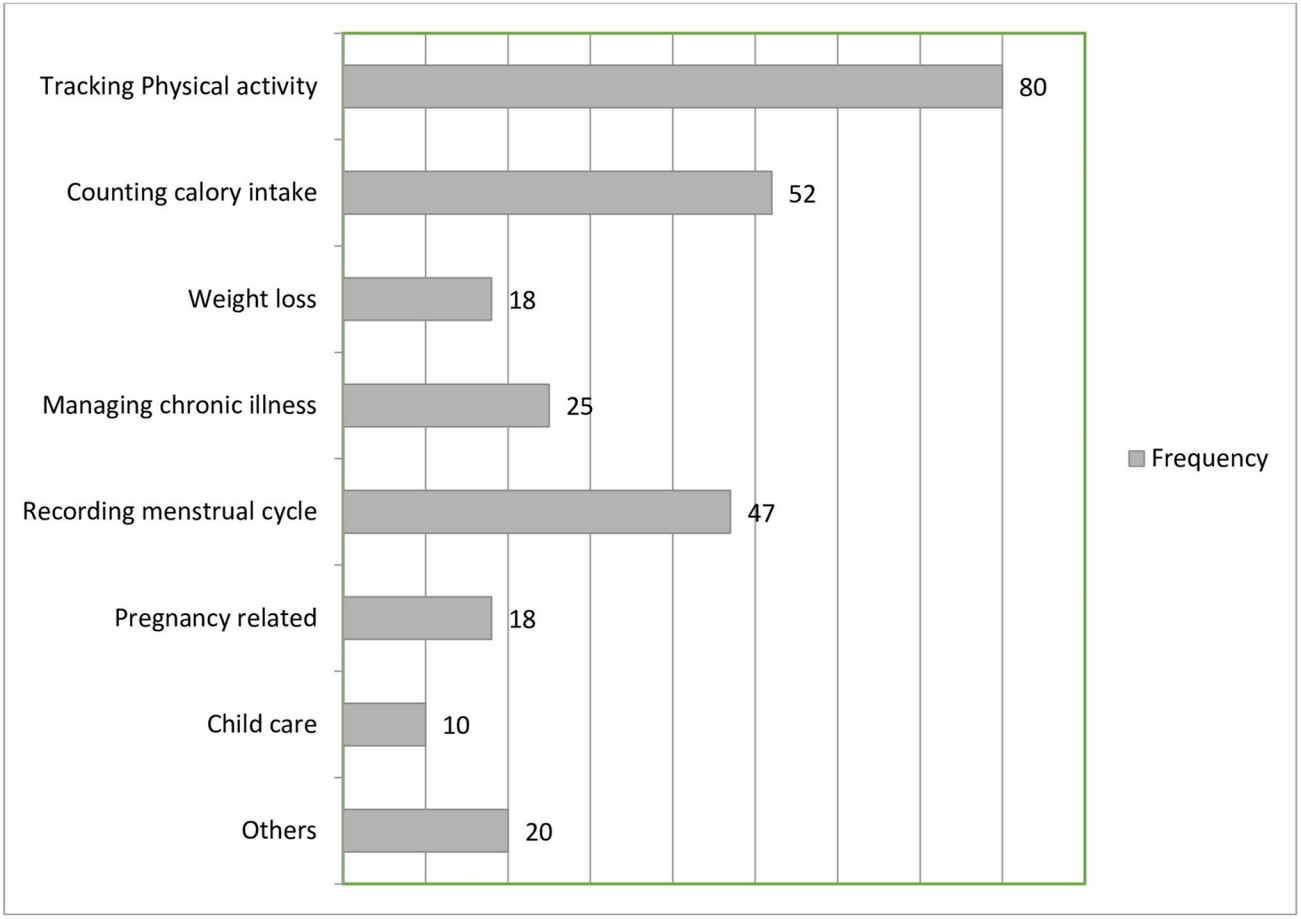
Purpose health related applications used by the students (N = 244). Note: A user can choose more than one application category.

Students were asked to choose the purpose health related applications installed on their smartphone from eight categories shown in the figure below, 32.8% (80/244) of the students stated that they have used mobile applications to track physical activity, and 10.2% (25/244) used managing chronic illness related applications while 8.2% the subjects stated that the purpose the applications they use is out of the listed category Fig 2

According to this study, only 6.4% of the study participants paid for health-related applications, while 88% of the study participants used freely available applications. On the other hand, 5.65% of the students didn’t remember whether they had paid for specific applications or not. Most of the students, 73.4% prefer health applications in the English language and 9.4%prefer applications in the Amharic language, while the remaining 17.2%used applications in both languages.

**Fig 3:**
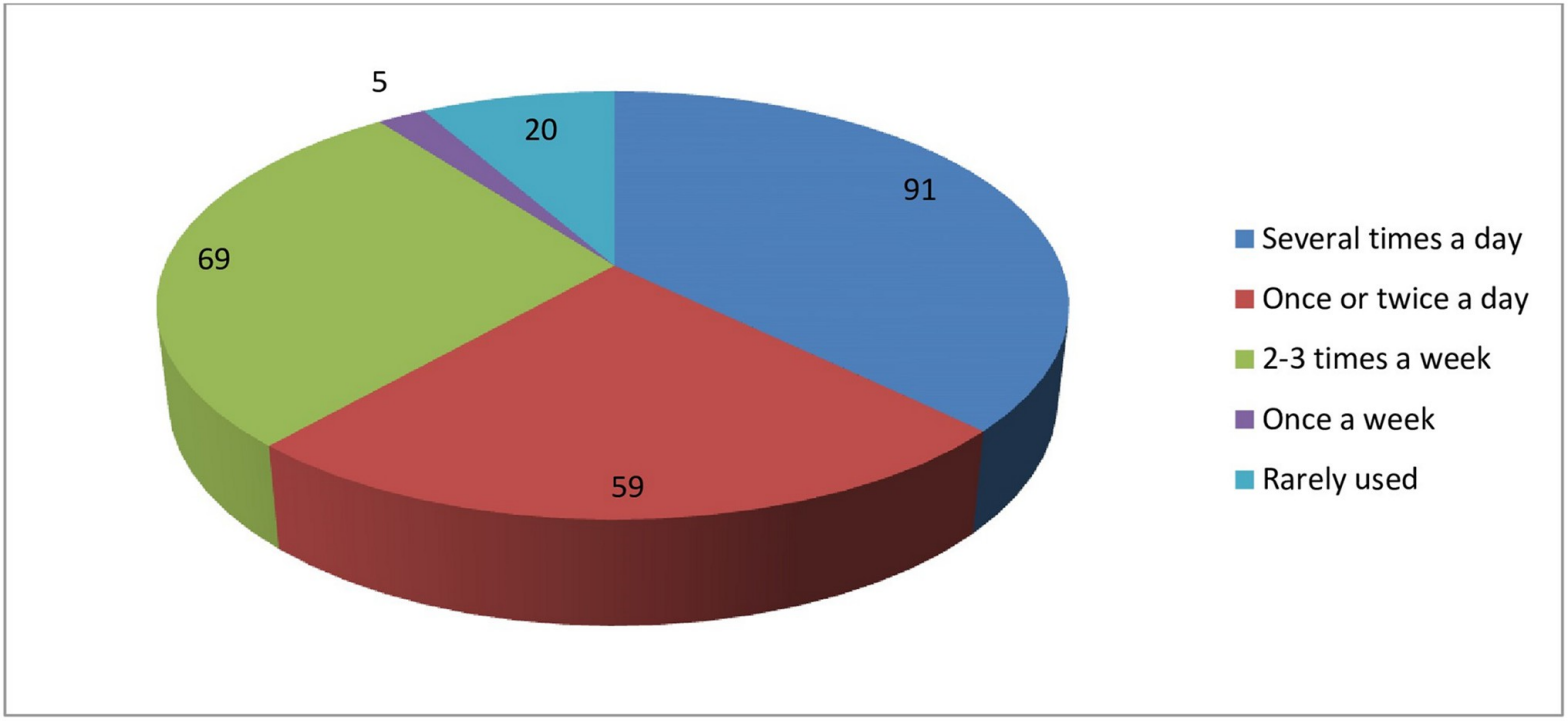
Frequency of health application usage (N = 244).

Self-reported frequency of application usage shows that 38.6% of the students use it several times a day, while 2.1% of the students use health applications rarely Fig 3. The daily time spent using these applications range from 1 minute to an hour; about half 48.4% of the students spend from 1–10 minutes a day using health application on their phone, while only 1% of the students use their applications for an hour. The average daily usage is 9 minutes a day. Usage history shows that 39.8% of the students have used between 0–6 months, the majority of the students 40% used the applications for a year, while 15.4% of the students utilized and used them for more than 2 years. On the other hand, 3.8% of the study participants didn’t remember the period they had been using the applications.

Participants were asked whether mobile health applications could reduce medical expenses and improve self-health management. The result indicated that 88% of the study participants agreed that mobile applications reduced medical costs and personal health management, 10% the students were unsure, and 2% said that health applications cannot reduce medical expenses.

**Fig 4:**
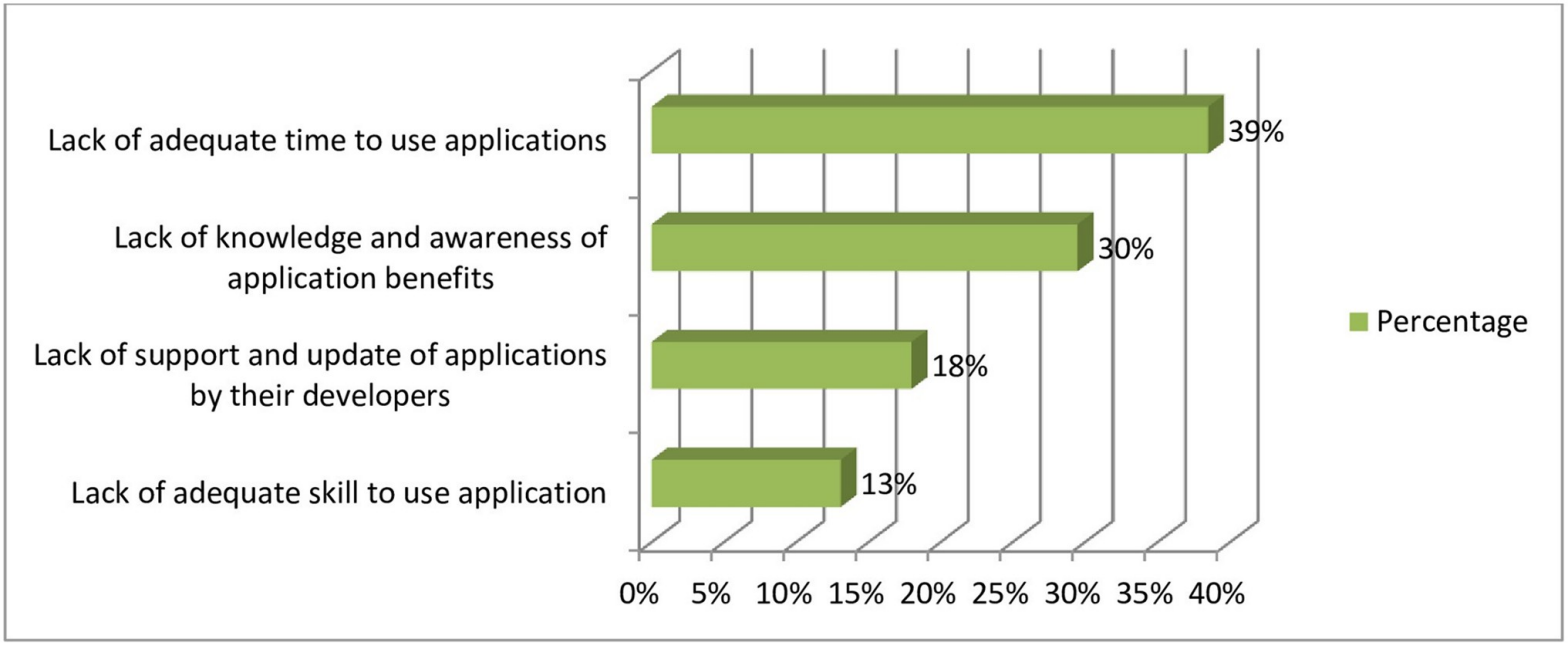
Barriers that could hinder mobile health applications among health science students (N = 244).

The study revealed that the most useful feature of the health applications reported by the students is accessibility. Almost half (52.5%) of the students stated that health applications are most easily accessible source of information in self-health management. On the hand students asked their to choose from list the barriers that could hinder the use of mobile applications, the most pertinent barriers reported were Lack of knowledge and awareness of application benefits, and Lack of adequate time to use applications Fig 4.

### Factors affecting mobile health application utilization among health science students

Various studies have documented the factors that influence the use of mobile health applications among users. In Ethiopia, this research has identified several determinants that impact the utilization of mobile health applications among health science students. Following bivariate logistic regression, six variables (easiness of health applications, language of applications, lack of support and updates, skill, level of study, age, and trust) were considered for multivariable logistic regression analysis. After conducting the analysis, four variables were found to be significantly associated with the utilization of mobile health applications among health science students Table 2. According to a multivariable logistic regression analysis, students are significantly more likely to use health applications that have a user-friendly interface and are easy to navigate (AOR = 4.8, 95%CI: [2.1–11.2]). Additionally, age is a determining factor in the utilization of mobile health applications among students, with those over 25 years old being twice as likely to use such apps than the other age groups (AOR = 2.01, 95%CI: [1.9–8.2]). Finally, a student’s technical ability to use health applications is also a determinant factor, as those who have this skill are 4.2 times more likely to use mobile health to manage their health (AOR = 4.2, 95%CI: [3.8–12]).

**Table 2:** Factors associated with utilization of mobile health application among health science students (N = 405).

Variables	Category	m-Health utilization		Crude OR^a^ (95% CI)	p-value	AO R^b^ (95% CI)	p-value
		Yes	No				
Age							
	Under 20	27	31	1		1	
	20–25	179	120	1.7(.9–3)	.062	1.31(1.9–7.2)	.035
	Above 25	40	6	7.7(3–20)	.000	2.01(1.9–8.2)	.011
Easiness							
	Yes	224	104	5.1(3–8.9)	.000	4.8(2.1–11.2)	.000
	No	22	53	1		1	
Skill							
	Yes	221	97	5.5(3.2–9.2)	.000	4.2(3.8–12)	.000
	No	25	60	1		1	
Trust							
	Yes	46	44	2.8(1.6–4.8)	.001	2.5(1.7–6.8)	.000
	No	40	108	1		1	
Lack of support							
	Yes	68	86	.31(.21-.48)	.001	.15(.06-.32)	.001
	No	178	71	1			

**OR**^**a**^: odds ratio

**AO R**^**b**^: Adjusted odds ratio

## Discussion

This study was conducted to determine mobile health utilization among health science students. Despite a large number of students owning smartphones, only 60.5% (95% CI: [56.6–65.5]) of health science students utilize mobile health applications, according to this study. This result is similar to a study conducted among pharmacy students in Istanbul Turkey [38], but higher than the study carried out at Jazan University in Saudi Arabia [17]. The variation could be due to differences in sample size and time gap between the studies. The study examined the most commonly used smartphone operating system, type of mobile health application, and frequency and duration of usage. In contrast to previous studies that report IOS as the most commonly used smartphone type [39]; only 12% owned IOS phones in this study. About three-fourths (74%) of participants owned android phones, which could be attributed to the high cost and inaccessibility of iPhones in the Ethiopian market. Participants were asked to select categories for their mobile health applications and list their names for each category. The results showed that physical activity tracking applications were the most commonly used, followed by calorie intake tracking applications, while childcare applications were least used. Although there was little difference in some categories compared to previous studies [17]. Samsung Health and Fitness, Jefit, Medscape, Strava, Fitbod, CLUE, Eve Tracker, and Liyana Care were among the most commonly used specific health applications.

The study evaluated the history of application usage among students to determine the duration of their use of smartphone applications. Results showed that 40% of students have been using mobile health applications for over a year, while 15.4% have used them for more than two years. These findings are consistent with previous studies [17, 26, 40, 41].

However, when asked why they do not download or use health applications, respondents cited lack of trust, complexity of use, cost, and lack of recommendation from teachers and healthcare professionals as the most common reasons. Interestingly, a previous study found that cost was not a barrier for students [17], which may be due to differences in economic status and availability of online payment methods in the study area. Additionally, respondents reported other barriers such as lack of knowledge and awareness about the benefits of using health applications and insufficient time to use them.

An investigation was carried out to explore various factors linked to mobile health applications and their connection with the outcome variable. The findings indicated that students’ technical proficiency was a significant factor in determining the utilization and usage of mobile health. Students possessing technical skills were 4.2 times more likely to use mobile health apps for managing their health compared to those without such skills (AOR = 4.2, 95%CI: [3.8–12]). Although a limited study was conducted in this area for comparison, similar results were reported in related studies conducted among healthcare professionals [24]. As users are increasingly concerned about privacy, security, and confidentiality issues related to accessing health information, they tend to prefer trusted applications that safeguard their data. Therefore, trust emerged as another predictor variable associated with the utilization of mobile health applications. This finding is consistent with previously published research conducted at Iran University of Technology [38, 40].

## Conclusion and recommendation

The study aimed to assess the extent of mobile health usage among health science students, revealing moderate usage. It also identified factors and barriers affecting mobile application use. Developers and healthcare planners should work towards promoting facilitating conditions and overcoming barriers to enhance mobile health utilization as education and preventive health strategy for the younger generation. Understanding the determinants of utilization can help healthcare professionals tailor interventions to promote technology use in healthcare settings. The study provides valuable insights into how mobile health applications can improve healthcare outcomes in low-resource settings like Ethiopia. Furthermore, for future researchers, we suggest using advanced models like the revised UTAUT model to evaluate various behavioral and technological factors that influence mobile health utilization.

### Limitation

This study’s limitation was that it was conducted at a single institution within a health science college. To obtain more generalizable results, incorporating various colleges and institutions would have been beneficial.

## Supporting information

S1 Data (SAV)
